# Measuring Parathyroid Hormone (PTH) in Patients with Oxidative Stress – Do We Need a Fourth Generation Parathyroid Hormone Assay?

**DOI:** 10.1371/journal.pone.0040242

**Published:** 2012-07-06

**Authors:** Berthold Hocher, Franz Paul Armbruster, Stanka Stoeva, Christoph Reichetzeder, Hans Jürgen Grön, Ina Lieker, Dmytro Khadzhynov, Torsten Slowinski, Heinz Jürgen Roth

**Affiliations:** 1 Institute of Nutritional Science, University of Potsdam, Potsdam-Rehbrücke, Germany; 2 Immundiagnostik AG, Bensheim, Germany; 3 Department of Nephrology, Charité-Mitte, Berlin, Germany; 4 Department of Endocrinology/Oncology, Limbach Laboratory, Heidelberg, Germany; UAE University, Faculty of Medicine & Health Sciences, United Arab Emirates

## Abstract

Oxidation of PTH at methionine residues results in loss of biological activity. PTH may be oxidized in patients with renal disease. The aim of this study was to develop an assay considering oxidation of PTH. Oxidized hPTH was analyzed by high resolution nano-liquid chromatography coupled to ESI-FTT tandem mass spectrometry (nanoLC-ESI-FT-MS/MS) directly and after proteolytic cleavage. The oxidized hPTH(1–84) sample shows TIC-peaks at 18–20 min and several mass peaks due to mass shifts caused by oxidations. No significant signal for oxidized hPTH(1–84) species after removal of oxidized PTH molecules by a specific column with monoclonal antibodies (MAB) raised against the oxidized hPTH was detectable. By using this column in samples from 18 patients on dialysis we could demonstrate that measured PTH concentrations were substantially lower when considering oxidized forms of PTH. The relationship between PTH concentrations determined directly and those concentrations measured after removal of the oxidized PTH forms varies substantially. In some patients only 7% of traditionally measured PTH was free of oxidation, whereas in other patients 34% of the traditionally measured PTH was real intact PTH. In conclusion, a huge but not constant proportion of PTH molecules are oxidized in patients requiring dialysis. Since oxidized PTH is biologically inactive, the currently used methods to detect PTH in daily clinical practice may not adequately reflect PTH-related bone and cardiovascular abnormalities in patients on dialysis.

## Introduction

Secondary hyperparathyroidism occurs frequently in chronic kidney disease as an adaptive response to deteriorating renal function. A combination of factors contribute to the increase of PTH that are additive [1]. Circulating 1,25-dihydroxy vitamin D starts to decrease very early in stage 2 of chronic kidney disease and continues to fall as the glomerular filtration rate (GFR) decreases further, and the renal 1α-hydroxylase is inhibited by hyperphosphataemia, hyperuricaemia, metabolic acidosis as well as 25-hydroxyvitamin D deficiency [1]. As GFR decreases below 60 mL/min/1⋅73 m^2^, phosphate is retained and stimulates synthesis and secretion of PTH. Hypocalcaemia develops as the GFR decreases below 50 mL/min/1⋅73 m^2^, further stimulating release of PTH. With disease progression, intact PTH (1–84) half-life increases and C-terminal fragments of the hormone accumulate. A relative state of end-organ resistance to the hormone exists, but chronic elevation of it has major consequences resulting in bone loss (particularly cortical bone), fractures, vascular calcification, cardiovascular disease, and hence an increased cardiovascular mortality [1].

A reliable method to measure PTH is a key for detecting patients with hyperparathyroidism as well as subsequent monitoring of therapeutic interventions. Therefore big efforts have been made in the past decades to develop PTH assays that are suitable for clinical use. So far three generations of PTH assays were developed as outlined below.

The first radioimmunoassay (RIA) for PTH was developed in the early 1960’s [2]. This assay used polyclonal sera from guinea pigs and rabbits directed against extracted bovine PTH. Cross-reactivity with human PTH was relatively low, allowing only for measurement of elevated PTH levels (e.g., patients with primary hyperparathyroidism), but not normal to low levels of PTH. About 10 years later, Arnaud and colleagues at the Mayo Clinic published their results measuring PTH with an improved RIA using an antiserum raised against porcine PTH [3]. This assay allowed measurement within the normal range because of the better cross-reactivity of the antibodies against porcine and human PTH.

When the full structure of the 84–amino acid PTH molecule was determined [4], it became obvious that most of the anti-PTH antibodies used at this time were directed against the carboxyl-terminus of the peptide. The biologic activity of the hormone, however, is located in the amino-terminal residues of the molecule and a major cleavage site in the PTH molecule was identified at residues 33–34 Thus, these PTH assays detected the biologically active 84–amino acid PTH molecule, but also biologically inactive carboxy-terminal breakdown products of PTH. This is especially a problem in patients with renal failure, since inactive carboxyl-terminal PTH fragments accumulate in these patients, leading to falsely elevated results in the assay.

A second-generation assay was described in 1987 [5]. This PTH assay claimed to measure “intact PTH”, since it used a pair of affinity purified antibodies, specific for two different regions of the PTH molecule. The capture antibody was directed against the carboxyl-terminal part of the hormone (amino acids 39–84), whereas the detection antibody was specific for the amino-terminal one (amino acids 1–34). PTH was sandwiched between the two antibodies. Only “intact” hormone molecules were detected. In addition to the increased specificity, such “two-site” assays were recognized as providing increased analytical sensitivity.

Third generation PTH assay was developed when it became apparent that patients with renal failure sometimes have intact PTH levels out of proportion to the level of bone disease even when 2^nd^ generation assays were used [6]. At the time, it was suggested that the discrepancy was caused by the presence of a large PTH fragment lacking residues 1–6 [7]. Such a fragment would be expected to bind to PTH receptors but would lack biological activity. It was suggested but never proven that these species represent up to 50% of PTH detected in uremic patients [7]. This has led to the development of 3^rd^ generation assays. They use the same carboxyl-terminal capture antibodies as the 2^nd^ generation assays but the detection antibodies are specific for amino acids 1 to 4. However, even these 3^rd^ generation tests have not improved the diagnosis of bone disease or other clinical manifestations of secondary hyperparathyroidism in uremic patients. Furthermore, there appears to be no difference in the diagnostic sensitivities between 2^nd^ and 3^rd^ generation assays in the diagnosis of primary hyperparathyroidism in patients with normal renal function [8]. Thus, it is so far not clear whether the initial hypothesis that led to the development of the 3^rd^ generation PTH assays is valid or not. It remains still unknown why in particular in uremic patients there is often a discrepancy between bone disease and measured PTH levels.

**Figure 1:**
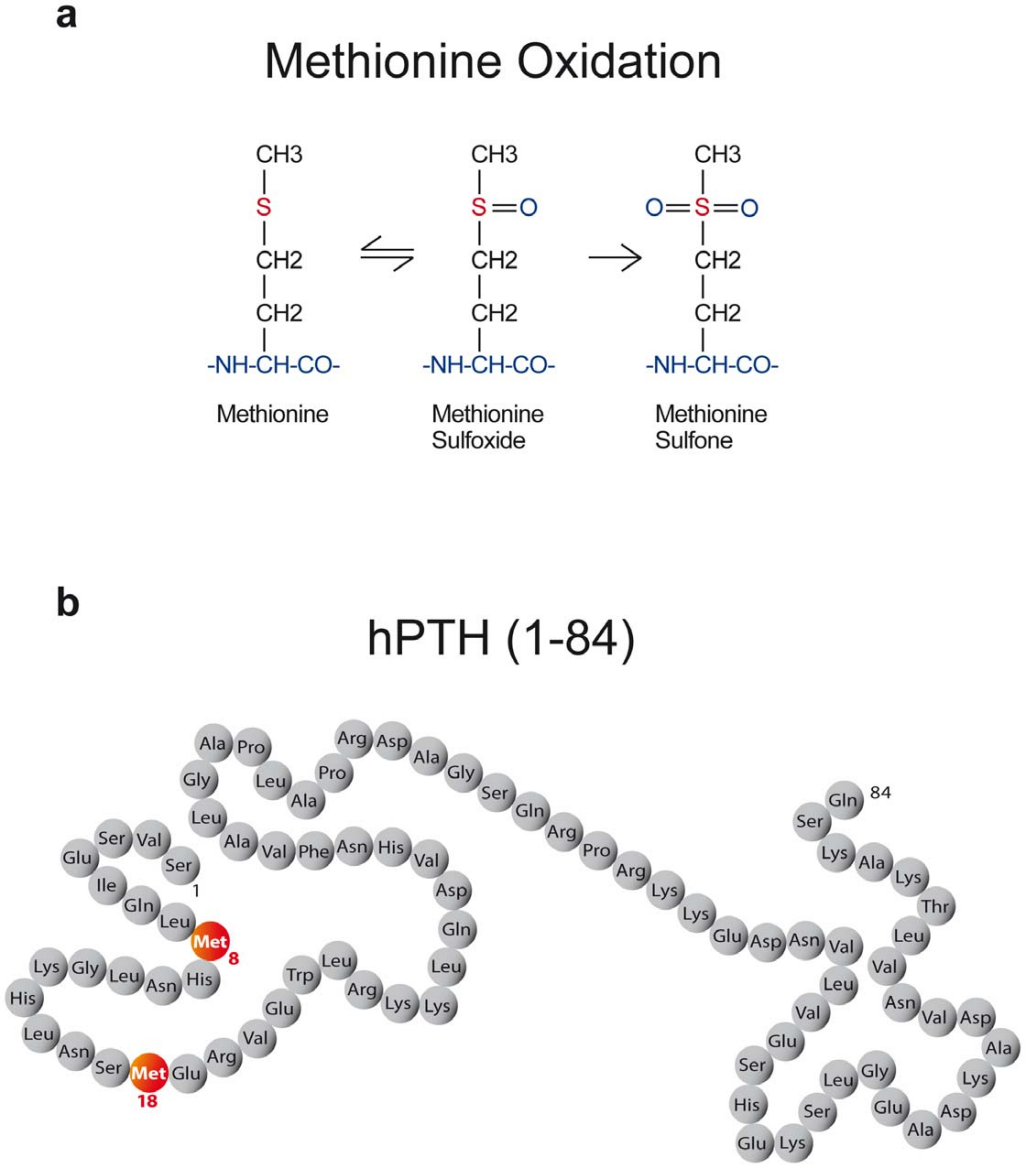
A: Under conditions of oxidative stress the methionine residues at position 8 and 18 may be oxidized to methionine sulfoxide and methionine sulfone. Oxidation to methionine sulfoxide is reversible, whereas the second oxidation step to methionine sulfone is irreversible. Oxidized PTH changes its 3-dimesional structure. This blocks the interaction of PTH with its receptor. B: Schematic diagram of the full length PTH(1–84) molecule (“bioactive” intact PTH). Oxidation at position Met 8 and/or Met 18 (red) alters the receptor binding site of PTH. Oxidized PTH does not bind the PTH receptor anymore and is thus biologically inactive. (see: E. Blind, Clin. Lab. 2008;54∶439-446, reference 3).

Patients with more advanced stages of renal disease are subject to oxidative stress; accordingly hormones like PTH are oxidized. The oxidized hormones may lose their biological activity by losing their ability to interact properly with their receptors. PTH has two methionine residues at position 8 and 18 (see figure 1), and indeed studies by independent groups have repeatedly shown that oxidation of PTH diminishes its interaction with its receptor. Oxidized PTH (Figure 1) does not stimulate the PTH receptor to generate cAMP, and is thus most likely biological inactive [9]–[12]. These studies showed clearly on a cellular level measuring the second messenger and on whole animal approaches measuring calcium, phosphorus and vitamin D that oxidized PTH loses its biological properties. In the current study we describe the development of an assay that is able to distinguish between oxidized PTH and biologically active PTH. We furthermore used this assay in a patient population known to be exposed to oxidative stress: end-stage renal disease patients on intermittent hemodialysis [13].

## Methods

### Oxidation of hPTH(1–84)

Human PTH(1–84) was purchased from Bachem (Bubendorf, Switzerland). 200 µg hPTH(1–84) were dissolved in 400 µl of 0.1 M acetic acid (final concentration of 0.5 µg/µl), mixed 1∶1 with 30% hydrogen peroxide and incubated for 45 min at 37°C. Afterwards, the mixture was cooled on ice, divided into aliquots and lyophilized.

Specificity test of anti-human oxidized PTH monoclonal antibody. The antibody was developed by Immundiagnostik AG, Bensheim, Germany. Based on the previously published methods [14], we now selected hybridoma cell lines from previously generated cell lines (14) producing antibodies against all forms of oxidized parathyroid hormone.

In order to characterize the specificity of the monoclonal antibody (MAB) raised against oxidized human PTH fragments, the antibody was immobilized on CNBr-activated Sepharose 4B (GE Healthcare Bio-Sciences, Uppsala, Sweden). Hundred µl aliquot of the slurry was filled in a column (MobiSpinColumn, MoBiTec, Göttingen, Germany) and equilibrated with PBS buffer, pH 7.4. Then 2.5 µg of lyophilized oxidized hPTH(1–84) were dissolved in 300 µl of equilibrating buffer and applied on the column. The column was incubated end-over-end for 1 h at room temperature, washed with 300 µl of equilibrating buffer, followed by 3 washes with 300 µl of distilled water, and then eluted 2 times with 200 µl of elution buffer (0.1% TFA). Flow-through, wash fractions (equilibrating buffer and water) as well as eluate of the column were collected separately, lyophilized and analyzed by nanoLC-ESI-FT-MS.

**Figure 2:**
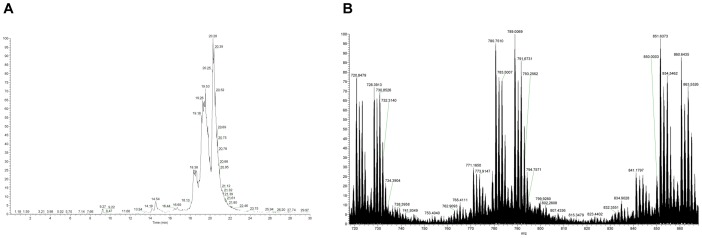
Non-digested oxidized synthetic hPTH(1–84)ox. A: NanoLC-ESI-FTMS total ion chromatogram. B: Magnified summed FTMS spectrum for retention time interval 18.30–20.50 minutes. Several different charged analyte ions were detected.

**Figure 3:**
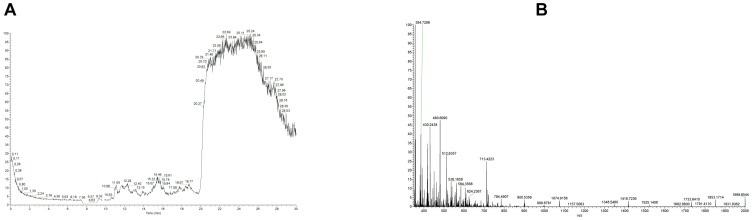
Flow through fraction of non-digested oxidized synthetic hPTH(1–84)ox from the affinity column. **A**: NanoLC-ESI-FTMS total ion chromatogram. **B**: Magnified summed FTMS spectrum for retention time interval of 16.50–18.50 minutes. The spectrum does not show any analyte masses which belong to PTH or oxidized PTH.

**Table 1:** Deduced molecular weights from the differently charged peaks in the spectra of the starting material, non-digested oxidized synthetic hPTH(1–84)ox (Fig. 1B), and the eluate from the affinity column (Fig. 3B).

Mass [m/z]	Charge z	MW [Da]	MW increase
728.16	13	9453.08	+32
729.39	13	9469.07	+48
730.62	13	9485.06	+64
731.85	13	9501.05	+80
780.50	12	9354.00	+32
781.83	12	9369.96	+48
783.17	12	9386.04	+64
788.76	12	9453.12	+32
790.09	12	9469.08	+48
791.42	12	9485.04	+64
792.75	12	9501.00	+80
851.36	11	9353.96	+32
852.82	11	9370.02	+48
854.27	11	9385.97	+64
860.28	11	9452.08	+32
861.73	11	9468.03	+48
863.19	11	9484.09	+64
864.64	11	9500.04	+80

The molecular masses correspond to values shifted by +16, +32, +48, +64 Da caused by methionine oxidation (sulfoxide, +16 Da and sulfone, +32 Da for each residue, and combinations thereof, maximal+64 Da) and by +80 Da for the additional oxidation of tryptophan 23.

### nanoLC-ESI-FT-MS/MS

In order to investigate the amino acid oxidation of human PTH(1–84), the sample was analyzed directly by high resolution nanoLC-ESI-FT-MS/MS to determine the masses of the whole molecule species and after proteolytic cleavage by three endoproteases (ArgC, LysC and chymotrypsin) to characterize the methionine oxidation at positions 8 and/or 18.

The non-digested samples were directly applied to nanoLC-ESI-FT-MS after acidification with 2% formic acid.

Before enzymatic digestion, the oxidized human PTH(1–84) sample (1 nmol) was denatured by 8 M urea containing 20 mM TCEP (tris[2-carboxyl]phosphine) reducing agent for 30 min. Iodoacetamide was added to 50 mM final concentration and the mixture incubated for another 20 min in the dark. After dilution to 0.8 M urea, the sample was digested separately by endoproteases (ArgC, LysC and chymotrypsin; enzyme to protein ratio (w/w): 1∶50) according to Proteome Factory’s protein digestion SOPs. The acidified peptide sample digests (ArgC, LysC and chymotrypsin) were pooled and applied to nano-LC-ESI-MS (LTQ-FT, Thermo Scientific) analysis using a 35 min nanoLC gradient (Agilent 1100 nanoLC system) with solvent A (0.1% formic acid/5% acetonitrile/94.9% ddH2O) and solvent B (0.1% formic acid/99.9% acetonitrile).

The mass accuracy was better than 5 ppm for MS data. The MS data were analyzed by MASCOT (Matrixscience) and Qualbrowser (Thermo Scientific) according to the predicted peptide masses.

**Figure 4:**
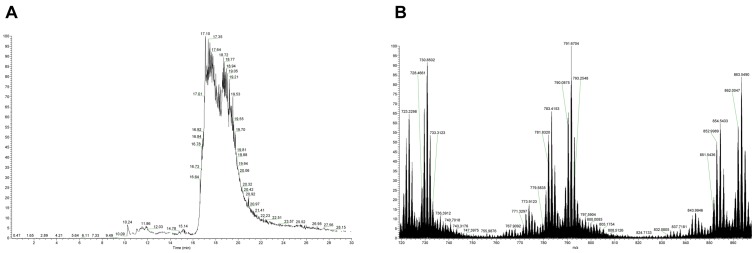
Eluate from the affinity column of non-digested oxidized synthetic hPTH(1–84)ox. **A**: NanoLC-ESI-FTMS total ion chromatogram. **B**: Magnified summed FTMS spectrum for retention time interval of 16.50–18.50 minutes. Several different charged analyte ions of PTH eluate were detected.

**Figure 5:**
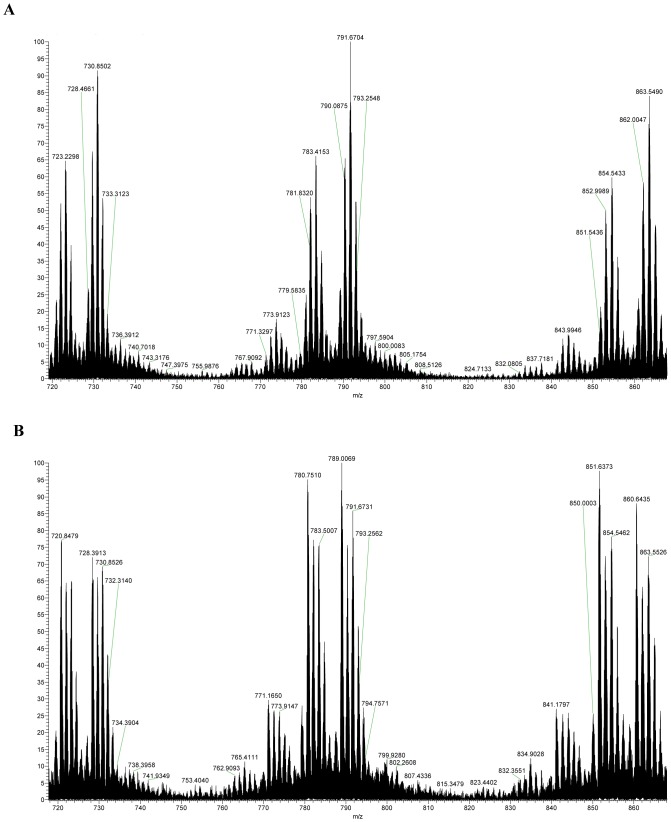
Comparison of the enlarged spectra of the starting material, non-digested oxidized synthetic hPTH(1–84)ox (**Fig.1B**), and the eluate from the affinity column of non-digested oxidized synthetic hPTH(1–84)ox (Fig. 3B).

### Blood Sample Preparation

For sample preparation, 100 µl aliquots of the slurry with the immobilized monoclonal antibody (MAB) were filled in MobiSpin-columns equilibrated with PBS buffer, pH 7.4. Then 500 µl of each sample were applied on the column, respectively. The columns were incubated mixing end-over-end for 2 h at room temperature, washed with 250 µl of 0.1 M ammonium acetate buffer pH 7.0, followed by a wash with 250 µl of 0.1 M ammonium acetate buffer pH 7.0, containing 20% acetonitrile, and then eluted 2 times with 200 µl of elution buffer (0.05 M formic acid, pH 3.5). Flow-through, wash fractions as well as eluate of the column were collected separately and lyophilized. Then the samples were reconstituted in 500 µl of PBS buffer, pH 7.4 and aliquots analyzed by the Roche Elecsys® PTH, Intact (Roche, Penzberg, Germany) assay, see below.

**Figure 6:**
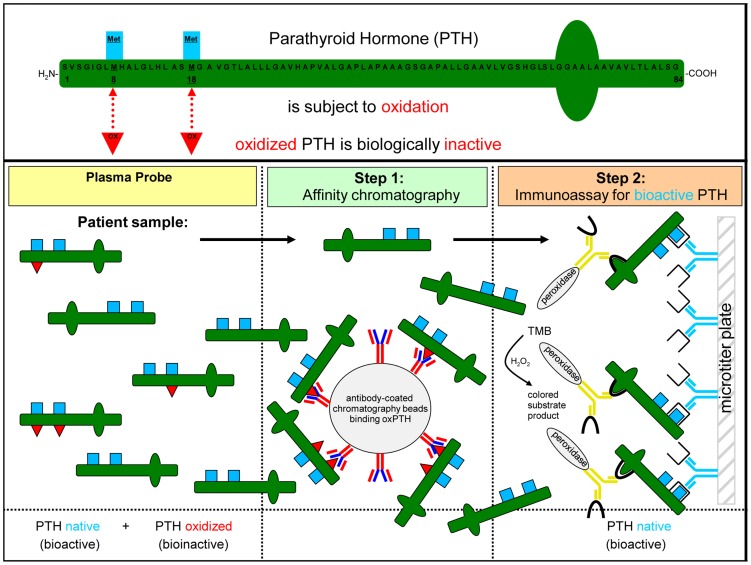
Basic principles of the new assay system for detection of intact and real intact PTH in human samples. The new detection process for real intact bioactive PTH consists of 2 steps: Firstly, any oxidized forms of oxidized PTH at position Met8 and/or position Met18 will be removed from the probe by a specific affinity chromatography column. The column contains monoclonal rat/mouse antibody (MAB) raised against the hPTH(1–34)oxidized fragments. These antibodies are able to remove any oxidized forms of PTH. In the second step the remaining non-oxidized PTH will be analyzed in conventional 2-site “sandwich” immunoassay systems. The antibody on the left (*capture antibody*) is bound to solid phase. The antibody on the right (*label antibody*) relays the signal. These antibodies must bind different sites on the PTH analyte to produce a positive result in the assay.

**Table 2:** Clinical Characteristic of the patients on dialysis.

No.	renal disease	Age(years)	Time on dialysis (years)	sex	iPTH	real-iPTH	ox-iPTH	Ratio iPTH/real-iPTH	Total Ca (mmol/l)	P (mmol/l)	CrP (mg/dl)
					(ng/L)	(ng/L)	(ng/L)				
1	Hypertensive Nephropathy	62	0,3	m	43,63	8,9	34,73	0.204	2,58	1,24	0,43
2	Diabetic Nephropathy	73	4	m	796,2	70,62	725,6	0.089	2,2	2,15	–
3	unknown	37	0,1	m	52,84	10,35	42,49	0.196	2,53	0,81	0,03
4	Diabetic Nephropathy	68	2,1	f	70,8	11,18	59,62	0.158	2,23	0,91	4,08
5	Acute Kidney Injury	64	0	m	46,49	9,45	37,04	0.203	2,17	1,32	3,26
6	Diabetic Nephropathy	63	1,6	f	42,13	5,37	36,76	0.127	2,08	1,43	12,2
7	ADPKD	70	3,3	f	1029	74,76	954,2	0.073	2,1	1,37	0,53
8	Cardio-Renal-Syndrom	70	3,4	m	240,4	41,89	198,5	0.174	2,38	1,57	0,32
9	unknown	70	9	m	105	18,48	86,52	0.176	2,26	1,5	3,12
10	Diabetic Nephropathy	65	7	m	1301	445,3	855,7	0.342	2,53	2,23	1,74
11	Membranous GN	45	5,4	f	311,8	24,44	287,4	0.078	1,57	2,06	0,52
12	Membranoproliferative GN(Typ1)	52	1,5	m	144,1	19,24	124,9	0.134	1,87	0,73	0,17
13	Hypertensive Nephropathy	61	4,1	m	73,45	15,92	57,53	0.217	2,15	2,35	0,67
14	ADPKD	57	1,2	m	281,9	44,02	237,9	0.156	2,18	1,35	13,4
15	Diabetic Nephropathy	73	4	m	116,9	19,73	97,17	0.169	2,38	1,66	4
16	Mesangioproliferative GN	69	8,1	m	70,81	18,51	52,3	0.261	2,62	2,28	6,7
17	interstitial Nephritis	61	2,6	f	76,28	11,21	65,07	0.147	2,21	1,61	2,9
18	unknown	56	10,6	m	487,1	76,12	411	0.156	2,35	2,41	0,17

**Figure 7:**
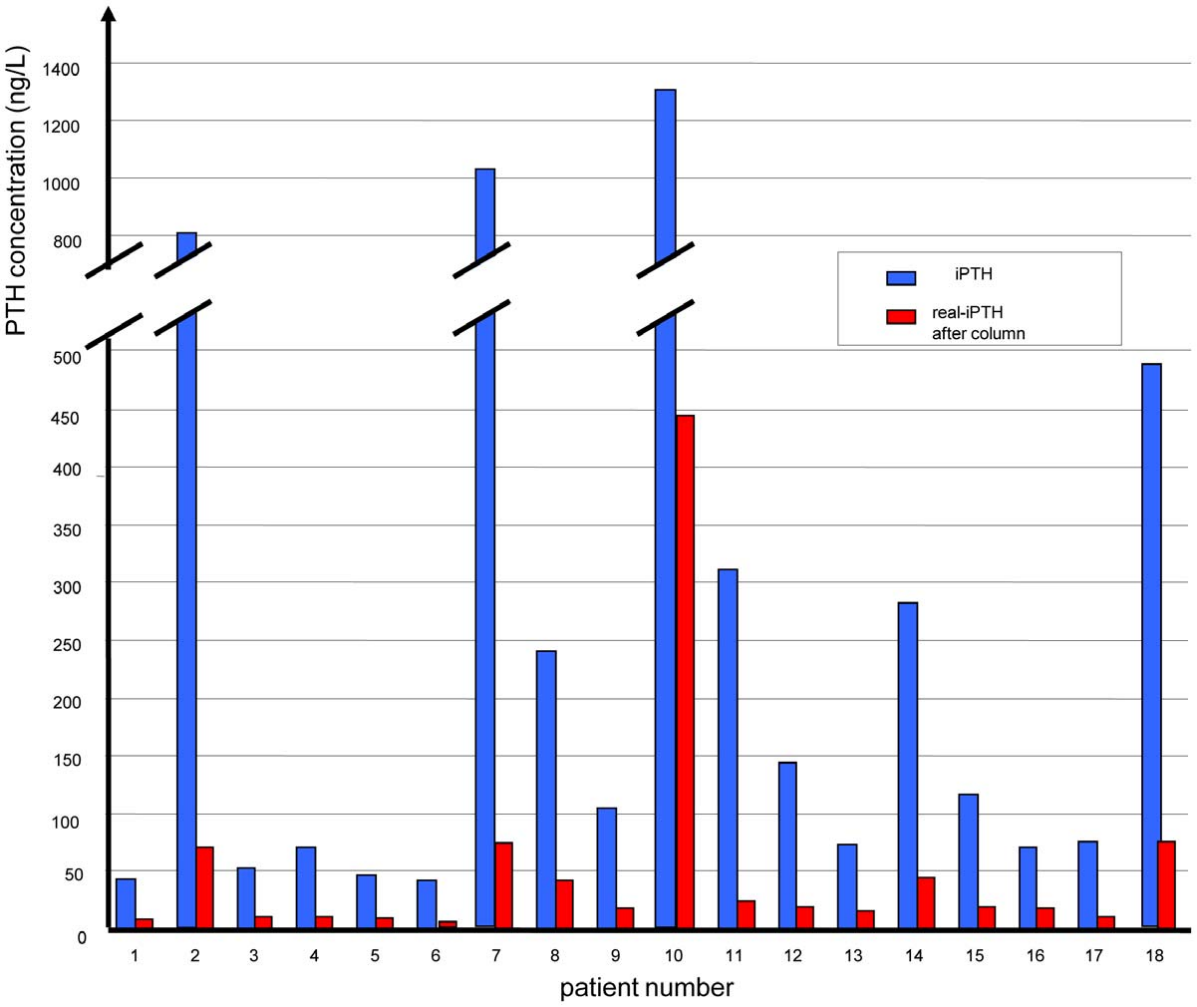
We measured intact PTH using the intact PTH assay as described in the method section in 18 patients on dialysis (blue bars), for further detail see also **table 2**. When removing the oxidized forms of PTH from the sample as illustrated in figure 6, the results were completely different (red bars). The effect of oxidation of PTH is highly variable among these patients requiring dialysis. There is only a very weak correlation between traditionally measured PTH and PTH data considering the oxidation of this hormone.

### Sample Spiking with PTHox

To prove the recovery of oxidized PTH, 500 µl of a sample were spiked with 1 ng oxidized PTH. The spiked samples were treated as described in the sample preparation part.

### Studying Clinical Specimens

We studied specimens from 18 patients on intermittent haemodialysis treated in our dialysis unit. Specimen (EDTA-whole blood) was taken just before the dialysis session started, centrifuged and stored immediately after plasma was obtained at −80°C until further analysis. The study was approved by the ethical committee of the university hospital Charité, Berlin, Germany. Written informed consent was obtained in each case. Patients’ characteristics were obtained from their clinical records. Serum phosphorus, calcium and C-reactive protein (CrP) were analyzed on an automatic analyzer of the clinical laboratory of the university hospital Charité.

**Table 3:** Control experiment: PTH was measured directly and after washing of the samples from a vitamin D column.

iPTH	iPTH after vitamin Dcolumn (ng/L)	Ratio
43,63	32,43	0,743295897
796,2	684,83	0,860123085
52,84	47,45	0,897993944
46,49	41,86	0,90040869
70,6	61,99	0,878045326

### Removal of Oxidized PTH from Human Specimens

500 µl of plasma samples were applied on the column with the immobilized monoclonal rat/mouse parathyroid hormone antibody (MAB). The column was exactly prepared as described above for the high resolution nanoLC-ESI-FT-MS/MS experiments.

The columns were incubated mixing end-over-end for 2 h at room temperature, washed with 250 µl of 0.1 M ammonium acetate buffer pH 7.0, followed by a wash with 250 µl of 0.1 M ammonium acetate buffer pH 7.0, containing 20% acetonitrile, and then eluted 2 times with 200 µl of elution buffer (0.05 M formic acid, pH 3.5). Flow-through, wash fractions as well as eluate of the column were collected separately and lyophilized. Then the samples were reconstituted in 500 µl of PBS buffer, pH 7.4 and aliquots analyzed by Roche Intact PTH assay, see below.

### PTH Assay

The intact-PTH electrochemiluminescence immunoassay (ECLIA; Roche PTH, Intact [iPTH]) uses a biotinylated monoclonal antibody, which reacts with amino acids 26–32, and a capture ruthenium-complexed monoclonal antibody, which reacts with amino acids 55–64. The determinations were performed on Roche Modular E 170®. The intraassay CV was 4.1% and the interassay CV was 5.8% at concentrations of 35.0 and 180.0 ng/L, respectively. Human samples were either measured directly (named iPTH) or after removal of oxidized PTH by a column with removing oxidized PTH using anti-hPTHox monoclonal antibodies.

### Control Tests

In order to be sure that the oxPTH columns remove specifically only oxidized parathyroid-hormone, we also analyzed some samples after purification with a column able to detect 1,25 dihydroxyvitamin D3 using vitamin D specific antibodies (Vit D-AssayK1107–737).

A second control experiment was designed to test the possibility that the monoclonal antibody (MAB) raised against the human oxidized PTH fragments may be released from the column and possibly interfere with the final PTH quantification: the conventional 2-site “sandwich” immunoassay system. We took samples from 2 patients and added high amounts of free monoclonal antibody (MAB) raised against the oxidized PTH fragments or solvent to the probes (the final concentration of the antibodies in the samples was 1.8 µg/ml). The samples were then analyzed by means of the iPTH immunoassay.

## Results

### nanoLC-ESI-FT-MS and nanoLC-ESI-FT-MS/MS Data

The intact hPTH(1–84)ox sample shows TIC-peaks at 18–20 min and several mass peaks due to +16, +32, +48, +64 and +80 Da mass shifts caused by the oxidations (methionine sulfoxide, +16 Da and sulfone, +32 Da for each methionine residue, and combinations thereof) (Fig. 2A,B).

The digested hPTH(1–84)ox sample shows oxidation of methionines 8 and 18 (methionine sulfoxide, +16 Da and sulfone, +32 Da for each methionine residue) in the analysis of the proteolytic peptides. Oxidation of tryptophan 23 (+16 Da) was observed additionally (data not shown).

No significant mass peaks were observed that can be assigned to any of the hPTH(1–84)ox species by nanoLC-ESI-FT-MS analysis of the flow-through and wash fractions (equilibrating buffer and water) of the column (Fig. 3A,B), whereas several mass peaks corresponding to the different oxidized states of hPTH(1–84)ox were detected in the eluate (Fig. 4A,B; Table 1).

Comparison of the spectra of the starting material, non-digested oxidized synthetic hPTH(1–84)ox (Fig. 2B), and the eluate from the affinity column of non-digested oxidized synthetic hPTH(1–84)ox (Fig. 4B) as illustrated in Fig. 5 reveals the same profile despite the difference in peak intensity.

The results demonstrate that the synthetic hPTH(1–84) was completely oxidized resulting in the formation of a variety of products corresponding to the different oxidized methionine status. In addition, the column with the monoclonal antibody (MAB) raised against the oxidized human PTH is specific for all oxidized forms of hPTH(1–84) and removed them all from the sample.

### Clinical Data

The clinical characteristics are shown in table 2. The method to detect oxidized and non-oxidized PTH is illustrated in figure 6. We included 17 patients on chronic hemodialysis as well as one patient requiring dialysis due to acute renal failure. We analyzed the clinical specimens with the iPTH immunoassay. In all patients the measured PTH concentrations were substantially lower when considering oxidized forms of parathyroid-hormone (see table 2 and figure 7). It is of note, however, that the relationship between PTH concentrations determined directly with the iPTH immunoassay and those concentrations measured after removal of the oxidized PTH forms is not constant, by contrast the relationship varies substantially probably due to the different degree of oxidative stress among the studied patients. In some patients only 7% of traditionally measured PTH were free of oxidation, whereas in another patient 34% of the traditionally measured PTH were real intact PTH. Taken together without considering oxidation status of PTH, the traditionally measured PTH concentrations using a modern sandwich detection system are severalfold higher as the concentrations when considering oxidation of PTH. The effect of oxidation of PTH is highly variable among these patients requiring dialysis. There is only a very weak correlation between traditionally measured PTH and oxidized PTH.

In some patients besides the iPTH immunoassay from Roche we also used the PTH(1–84) assay system from Roche. We basically got similar results as described above with the iPTH assay system. Without considering oxidation status of PTH, the traditionally measured PTH concentrations are detected severalfold higher as compared to the concentrations when considering oxidation of PTH (data not shown). This may indicate that all third generation PTH test systems (see introduction) used nowadays do not consider oxidation status of PTH.

In order to be sure that the oxPTH columns remove specifically only oxidized PTH, we also analyzed some samples after purification with a column able to bind 1,25 dihydroxyvitamin D3. These data indicate that only approximately 14% of the PTH is non-specifically bound by a “nonsense” (vitamin D) column. In other words, the column on its own when containing no antibodies raised against oxidized forms of PTH but antibodies detecting other epitopes does not significantly influence the test results (table 3).

A second control experiment was designed to test the possibility that the monoclonal rat/mouse antibody (MAB) raised against the hPTH(1–34)oxidized fragments may be released from the column and possibly interfere with final PTH quantification: the conventional 2-site “sandwich” immunoassay system (see figure 6). We took samples from 2 patients and added high amounts of free monoclonal antibodies (MAB) raised against oxidized human PTH fragments or solvent (the final concentration of the antibodies in the samples was 1.8 µg/ml). The samples were then analyzed using the iPTH immunoassay. Those samples where only solvent was added had measured iPTH concentrations of 43.63 [ng/L] (patient a), and 796,20 [ng/L] (patient b), respectively. Adding the monoclonal antibodies to the samples did not alter the results significantly. In the samples with antibodies we measured 35,70 [ng/L] (patient a) and 753,20 [ng/L] (patient b). This indicates that even in the case that the column may lose monoclonal antibodies (MAB) raised against the oxidized human PTH molecules, these antibodies do not interfere significantly with the final iPTH quantification (see figure 6).

## Discussion

Using very sensitive mass spectroscopy approaches, the current study clearly demonstrated that oxidation of human PTH(1–84) resulted in the formation of a variety of products corresponding to the different oxidized methionine resides at position 8 and/or 18 within the parathyroid hormone. A column with the monoclonal antibody raised against the hPTH(1–34)ox fragment is specific for all oxidized forms of hPTH(1–84) and removed them all from the sample. The clinical part of our study demonstrated that without considering oxidation status of PTH, the traditionally measured PTH concentrations based on current gold standard methods resulted in much higher PTH concentrations in the clinical samples as compared to the concentrations when considering oxidation of PTH. The effect of PTH oxidation is highly variable among patients requiring dialysis. There is only a weak correlation between traditionally measured PTH and PTH data considering the oxidation of this hormone. Given the fact that oxidized PTH (Figure 1) does not stimulate the PTH receptor anymore to generate cAMP, and is thus most likely biologically inactive [9]–[12], clinical strategies for the treatment of hyperparathyroidism in dialysis patients based on measurements of PTH using classical third generation sandwich ELISA techniques are most likely prone to incorrect decision making.

It is known for example that in uremic patients highly specific assays have measured a 2.5-fold increase in the non-suppressible fraction of PTH compared with healthy subjects [15]–[20]. Moreover, PTH concentrations measured in uremic serum apparently overestimated PTH-related bone abnormalities also by a factor of 2–2.5 [6]. It was suggested that in patients with chronic renal failure, the presence of high circulating levels of non-1–84 PTH fragments (most likely 7–84 PTH) detected by the second generation assay and the antagonistic effects of 7–84 PTH on the biological activity of 1–84 PTH may explain this [21]. However, this hypothesis was never proven in adequately designed clinical studies using for example HPLC coupled to mass spectrometry to really distinguish between different PTH fragments. Our data on the other hand using modern liquid chromatography linked to tandem mass spectroscopy to detect PTH suggest that this well-known overestimation of PTH in patients on dialysis might be most likely due to the presence of oxidized, biologically inactive forms of PTH in patients on dialysis.

Reactive oxygen species (ROS) such as hydrogen peroxide (H_2_O_2_) or hypochlorous acid (HOCl), and free radicals such as hydroxyl radical (OH) or others are continuously formed *in vivo*. Additional imbalance between formation of ROS and potent antioxidative defense mechanisms creates oxidative stress. Uraemia in general is associated with enhanced oxidative stress, and haemodialysis or peritoneal dialysis may in particular contribute to oxidative stress and reduced antioxidant levels in such patients [6]–[10].

One of the preferred highly sensitive targets for oxidation is methionine. The oxidation product methionine sulfoxide can be reversed by reduction with chemicals or biologically, whereas oxidation to the methionine sulfone is biologically irreversible (figure 1). Oxidation of methionine residues can lead to an activation or inactivation of a functional protein, respectively, and the resulting methionine sulfoxide can be reversed enzymatically by a specific reductase. Methionyl sulfoxide reductase has been found in *E. coli* and in mammalian tissues. Oxidation of methionine and its reversal may serve as a regulator for protein activities [12]. The parathyroid hormone contains two methionine residues in the amino-terminal region (position 8 and 18), responsible for the biological activity of the peptide, accessible to alterations by oxidation. Galceran et al. concluded in 1984 that oxidation of bPTH-(1–34) results in loss of both the renal and skeletal effects of PTH *in vivo* in rats and dogs [10]. Horiuchi postulated in 1988 that intact methionine residues at position 8 and 18 of hPTH-(1–34) are necessary for all its major biological actions, including its effect on the renal metabolism of 25-hydroxyvitamin D_3_ [11]. The secondary structure of the parathyroid hormone seems to be essential for its receptor binding. The methionine residue 8 is important for the folding of the hormone and proves the key role for this residue in the structure of the amino-terminal domain and its biological activity [12]. Thus oxidation of methionine residue 8, producing fundamental chances in secondary structure of PTH, is implicated both in binding and in activation of adenylyl cyclase. As early as 1974 O’Riordan and his group showed the effect of oxidation and back-reduction on the potency of porcine PTH, measured by its activation of rat renal cortical adenyl-cyclase [22].

Based on published data and our results, we suggest that methionine residues in different peptide hormones, like human growth hormone, somatomammotropin, luteotropin as well as PTH may be subject to oxidation resulting in loss of biological activity or receptor affinity. Methionine oxidation may be a general principle in regulation of hormone activity. However, this hypothesis needs to be proved in detail.

Our new assay system is - for the first time - able to differentiate between oxidized and non-oxidized forms of PTH by removing oxidized PTH fragments with a highly specific antibody able to detect and bind all forms of oxidized PTH. The removal of oxidized forms of PTH can be done either – as it was done in the present study – prior to analysis by a coated column followed by a third generation PTH assays (for assay principle see figure 6) or even as an integrative part of a third generation sandwich immunoassay system. It should also be feasible to combine our approach [14] with modern techniques like liquid chromatography coupled to tandem mass spectrometry [23], [24] in clinical practice in the near future by immunocapture oxidized PTH fragments prior to LC-MS/MS. This will improve the diagnostic performance of LC-MS/MS PTH approaches.

In conclusion, by means of nanoLC-ESI-FT-MS/MS, we were able to demonstrate that oxidation of human PTH(1–84) resulted in the formation of a variety of products corresponding to the different oxidized methionine residues at position 8 and/or 18 within the parathyroid hormone. A column with a monoclonal antibody (MAB) raised against the oxidized human PTH fragment is specific for all oxidized forms of hPTH(1–84) and removed them all from the sample. Without considering oxidation status of PTH, the traditionally measured PTH concentrations based on current gold standard methods resulted in much higher PTH concentrations in clinical samples specimens as compared to the concentrations when considering oxidation of PTH. The effect of PTH oxidation is highly variable among patients requiring dialysis. Given the impact of vascular calcification in end-stage renal disease patients on morbidity and mortality [25] further adequately powered studies are needed to demonstrate that measuring whole PTH without “contamination” of oxidized PTH forms improves clinical decision making and better reflects PTH-related bone and cardiovascular abnormalities. Reliable measurements of biologically active PTH are clinically important. Treatment guidelines for patients on dialysis as well as in- and exclusion criteria into clinical trials are based on PTH concentrations. Given the poor clinical performance of currently available assay systems for PTH measurement [26], a recent review even suggested that reliance on PTH concentrations (measured by the classical test systems) alone is a dangerous substitute for the search for, and use of, more precise and reliable biomarkers [27]. We suggest that this is at least partially attributed to the neglection of the oxidation status of the hormone. However, as stated above, this clearly needs to be investigated in detail in suitable clinical studies.
